# Changes in Circulating MicroRNA Levels as Potential Indicators of Training Adaptation in Professional Volleyball Players

**DOI:** 10.3390/ijms25116107

**Published:** 2024-06-01

**Authors:** Dominika Podgórska, Marek Cieśla, Artur Płonka, Wojciech Bajorek, Wojciech Czarny, Paweł Król, Rafał Podgórski

**Affiliations:** 1 Department of Internal Diseases, Institute of Medical Sciences, College of Medical Sciences, University of Rzeszow, 35-310 Rzeszow, Poland; 2 Institute of Medical Sciences, College of Medical Sciences, University of Rzeszow, 35-310 Rzeszow, Poland; mciesla@ur.edu.pl; 3 Institute of Physical Culture Studies, College of Medical Sciences, University of Rzeszow, 35-310 Rzeszow, Poland; plonka77@op.pl (A.P.); wbajorek@ur.edu.pl (W.B.); wojciechczarny@wp.pl (W.C.); pkrol@ur.edu.pl (P.K.); 4 Department of Biochemistry, Institute of Medical Sciences, College of Medical Sciences, University of Rzeszow, 35-310 Rzeszow, Poland; rpodgorski@ur.edu.pl

**Keywords:** microRNA, professional athletes, training biomarkers

## Abstract

The increasing demand placed on professional athletes to enhance their fitness and performance has prompted the search for new, more sensitive biomarkers of physiological ability. One such potential biomarker includes microRNA (miRNA) small regulatory RNA sequences. The study investigated the levels of the selected circulating miRNAs before and after a 10-week training cycle in 12 professional female volleyball players, as well as their association with cortisol, creatine kinase (CK), and interleukin 6 (IL-6), using the qPCR technique. Significant decreases in the miR-22 (0.40 ± 0.1 vs. 0.28 ± 0.12, *p* = 0.009), miR-17 (0.35 ± 0.13 vs. 0.23 ± 0.08; *p* = 0.039), miR-24 (0.09 ± 0.04 vs. 0.05 ± 0.02; *p* = 0.001), and miR-26a (0.11 ± 0.06 vs. 0.06 ± 0.04; *p* = 0.003) levels were observed after training, alongside reduced levels of cortisol and IL-6. The correlation analysis revealed associations between the miRNAs’ relative quantity and the CK concentrations, highlighting their potential role in the muscle repair processes. The linear regression analysis indicated that miR-24 and miR-26a had the greatest impact on the CK levels. The study provides insights into the dynamic changes in the miRNA levels during training, suggesting their potential as biomarkers for monitoring the adaptive responses to exercise. Overall, the findings contribute to a better understanding of the physiological effects of exercise and the potential use of miRNAs, especially miR-24 and miR-26a, as biomarkers in sports science and medicine.

## 1. Introduction

The changes occurring in the body during the long-term training and adaptation mechanisms in athletes have become focal points of numerous studies. An increasing number of factors are being recognized for their potential to influence an athlete’s performance and capacity to train more effectively. Among these, research has shown that a high ratio of testosterone to cortisol strongly correlates with positive training and performance outcomes [1]. Furthermore, vitamin D deficiency has been linked to reduced immunity, negatively impacting muscle regeneration and bone health [2,3], while a decrease in triiodothyronine levels diminishes the adaptability to training and contributes to overload injuries [4]. Intense training is known to induce transient exercise-induced muscle damage, resulting in elevated creatine kinase concentrations in athletes [5], while chronic iron deficiency reduces erythropoiesis, thereby diminishing the body’s endurance [6]. Evaluating the regulation of antioxidant systems during training is also crucial for healthy adaptation and disease prevention. This can be achieved through the assessment of biomarkers of pro-oxidant and antioxidant activity [7,8,9]. The parameters of inflammation, such as C-reactive protein, particularly high-sensitivity C-reactive protein, and cortisol, have also been employed to gauge the exercise intensity and associated changes [10,11].

In the pursuit of understanding the factors influencing cellular, tissue, and organ adaptation to exercise, increasing attention is being directed towards epigenetic mechanisms as modulators of physiological adaptation [12]. The human genome comprises approximately 3 billion base pairs, with only 1–2% consisting of protein-coding gene sequences [13]. A substantial portion of the genome consists of non-coding DNA regions, which regulate gene activity and uphold genome stability. These non-coding fragments include promoters, enhancers, inhibitors, and insulators of gene expression, depending on their location and function [14]. This mechanism is also very important for organismal adaptation during and after exercise [15]. Genetic factors account for 50–60% of the traits related to activity and physical effort, while environmental influences encompass the remainder [16,17,18]. Epigenetic changes integrate these factors by reversibly influencing gene expression, resulting in phenotypic changes without altering the DNA sequence [19,20,21]. The epigenetic mechanisms include methylation, histone modification, as well as non-coding RNAs, and are influenced by environmental factors such as diet, smoking, and exercise [22,23]. Non-coding RNAs encompass long non-coding RNAs and small non-coding RNAs [24,25], with microRNAs (miRNAs) being prominent representatives of the latter group, containing short RNA sequences of approximately 18–24 nucleotides [26]. Over 2500 miRNAs have been identified in humans to date [27]. They regulate post-transcriptional gene expression by inhibiting protein translation or enhancing mRNA degradation, resulting in reduced expression levels of the target proteins [28,29]. Typically, miRNAs bind to complementary sequences within the 3′UTR of the target gene to initiate this process [26]. Individual miRNAs target multiple genes, and, conversely, each gene site can be regulated by numerous miRNAs [30,31]. MiRNAs are released into circulation and other body fluids in a highly stable cell-free form, rendering them excellent potential diagnostic or predictive biomarkers associated with specific conditions [32,33]. The advancements in miRNA research underscore their critical role in development, physiology, and metabolism [34,35,36]. It has been demonstrated that up to 60% of the protein-coding genes in the human genome are regulated by miRNAs at the translational level [37,38]. The miRNA expression in skeletal muscle fluctuates in response to atrophy [39], overload-induced hypertrophy [40], single bouts of exercise [41], and exercise training [42].

In our study, we focused on the impact of physical activity on the levels of selected circulating microRNAs (miR-22, miR-17, miR-125b, miR-24, miR-26a, and miR-93), as well as their association with well-known physical activity biomarkers, such as cortisol, creatine kinase (CK), and interleukin 6 (IL-6). We evaluated the levels of these extracellular miRNAs before and after the training period to identify the predictors of favorable or unfavorable responses to exercise. It is crucial to determine the miRNA expression patterns that reflect the exercise adaptations at the molecular level to monitor physical performance, prevent muscle injuries, and track recovery. Our study analyzed selected circulating miRNA biomarkers indicative of the quality of the physiological process of adaptive responses to exercise. To the best of our knowledge, this is the first study to elucidate the effect of structured training on the levels of the investigated circulating miRNAs.

## 2. Results

The body composition parameters of 12 female players are displayed in Table 1.

Taking into account the changes in the body composition parameters, it is evident that the preparatory training had a significant effect on nearly every parameter assessed. Additionally, there was a notable increase in endurance as measured by the VO2max parameter (41.8 ± 4.54 mL/kg/min at the baseline compared to 45.57 ± 4.69 mL/kg/min at the endpoint; *p*-value < 0.001).

The relative quantities of four miRNAs (miR-22, miR-17, miR-24, and miR-26a) were significantly different between the baseline and endpoint (*p* = 0.009, *p* = 0.039, *p* < 0.001, and *p* = 0.003, respectively). All of them exhibited a decrease in the expression level during the training period. Specifically, the expression of miR-22 decreased by approximately 30% compared to the baseline, miR-17 decreased by 34.3%, miR-24 showed a 44.4% lower expression value, and miR-26a exhibited 45.5% lower plasma levels. The detailed data are presented in Table 2. The concentrations of IL-6 did not differ between the baseline and endpoint (*p* = 0.22). We found a significant increase in the CK levels (*p* = 0.001). Surprisingly, the cortisol levels significantly decreased from 25.57 ± 7.45 µg/dL at the baseline to 16.66 ± 4.26 µg/dL at the endpoint (*p* < 0.001).

Throughout the entire training period, a correlation was observed between the miR-125b concentration and creatine kinase (r = 0.46). At the baseline, no correlations were observed between the miRNAs and laboratory markers (creatine kinase, cortisol, and IL-6), as well as the body composition parameters and VO2 max. However, at the endpoint, correlations between miR-24 and creatine kinase (r = 0.67) and BMI were found (Table 3). The detailed data are provided in the Supplementary File (Tables S1 and S2).

A multiple linear regression analysis was conducted to evaluate the potential impact of miR-22, miR-17, miR-24, and miR-26a on the levels of creatine kinase, cortisol, and VO2 max. The analysis revealed that creatine kinase was dependent on the mentioned miRNAs (Table 4). The regression analysis (R = 0.89; R2 = 0.8 and R2 (adjusted) = 0.69) demonstrated that miR-24 (*p* = 0.003) and miR-26a (*p* = 0.042) had the most significant impact on the creatine kinase concentration levels. The investigated microRNAs do not impact the levels of cortisol or VO2. The detailed data are provided in the Supplementary File (Tables S3 and S4).

A bioinformatic analysis was also conducted, revealing that miR-24 may potentially regulate 30 genes, while miR-26a may potentially regulate 12 genes (detailed lists of genes are provided in Supplementary Tables S5 and S6). Among these genes, the *MAPK14* gene, which encodes p38α, appears to be particularly intriguing in the context of the potential regulation of the creatine kinase concentration.

## 3. Discussion

The aim of our study was to investigate the impact of intense training on the levels of selected circulating extracellular microRNAs from plasma and their association with well-known physical activity biomarkers in professional female volleyball players. Our findings shed light on the dynamic changes in the levels of specific microRNAs, such as miR-17, miR-22, miR-24, and miR-26a, during a 10-week training cycle. All of them are associated with the body’s response to inflammation. We found a significant decrease in the levels of these miRNAs following the intensive training period. Additionally, we observed reduced levels of inflammatory biomarkers (cortisol and IL-6) at the endpoint compared to the start of training. These observations suggest a potential association between these microRNAs and the physiological adaptations occurring in reaction to the training stimulus, emphasizing their feasible role as molecular markers of adaptive responses to exercise. Particularly, the marked reduction in the miR-24 and miR-26a levels correlated with elevated creatine kinase concentrations, implying their potential role in modulating the muscle damage and repair processes. Additionally, the analysis of the body composition parameters demonstrated significant changes between the baseline and endpoint, indicating shifts in weight, fat percentage, fat mass, fat-free mass, total body water, basic metabolic rate, and VO2 max. Our findings suggest that the investigated miRNAs might serve as potential biomarkers reflecting the physiological adaptive response to exercise.

We investigated the circulating extracellular microRNAs in peripheral blood material, focusing on plasma samples. Plasma is an easily collectible biological material with a non-invasive procedure for obtaining it, and it can be stored long-term. Moreover, it is nuclease-free, particularly devoid of ribonucleases, ensuring stability against biological degradation. Additionally, most of the molecular procedures require only a small sample volume with high sensitivity and specificity for the polymerase chain reaction (PCR) method, making it an attractive method for identifying potential biomarkers [43]. Alternatively, the miRNA levels can be evaluated directly from the cells responsible for their expression, such as sub-populations of leukocytes from whole blood or cells from muscle tissue. The primary goal of this approach is cell identification, which can be achieved through sorting the direct population using techniques like flow cytometry or magnetic beads. However, these procedures are preferably performed on unfrozen cells, increasing the complexity, time, and cost of the analysis. From the perspective of our analysis, the cell-based methods are less attractive compared to searching for an extracellular marker [44,45]. 

A range of biomarkers have been identified as indicators of the body’s adaptation to exercise. One of them is IL-6. The role of IL-6 in adaptation to exercise has been extensively studied, and the evidence suggests that IL-6 plays a multifaceted role in the body’s response to exercise [46,47]. Interleukin 6 is a well-known cytokine that plays a crucial role in the body’s immune response to infections and trauma. It is produced by various cell types, including T cells and macrophages. While initially considered a pro-inflammatory cytokine, the role of IL-6 is complex and not fully understood. It has both pro-inflammatory and anti-inflammatory properties depending on the context and the microenvironment. The concentration of IL-6 increases up to 100-fold after a marathon race. Recent studies have demonstrated that IL-6 is produced locally in contracting skeletal muscles, and the overall release from the muscle can explain the exercise-induced rise in the arterial concentration. Larger quantities of IL-6 are produced in response to exercise compared to any other cytokine. IL-6 is locally produced in the skeletal muscle in response to exercise, and it is known to stimulate the hepatic glucose output and induce lipolysis [48]. However, the data on changes in the plasma IL-6 concentration during exercise are more inconclusive as the IL-6 excretion can vary depending on the type of exercise, intensity, and duration, as well as the form of contraction (e.g., eccentric or concentric) [48]. Other commonly utilized biomarkers for assessing muscle damage and fatigue are creatine kinase and cortisol. Cortisol, a catabolic hormone, has the potential to reduce protein synthesis while increasing protein degradation, potentially impeding the training adaptation. Conversely, the serum creatine kinase levels are closely associated with energy metabolism and can serve as an indicator for monitoring the training load and assessing the functional recovery [49]. However, it is important to note that the levels of cortisol and creatine kinase can be influenced by various factors, such as dietary components, stress, personal issues, and physiological conditions. Additionally, variables such as alcohol consumption, oral contraceptive use, age, marital status, and parity have been shown to significantly impact the creatine kinase levels [50]. Our study confirmed these findings, particularly regarding cortisol, whose levels were elevated at the study’s outset, even surpassing the normal range. This phenomenon can likely be attributed to the stress associated with returning to the club and potential fatigue following the journey. Given the multitude of factors that can influence traditional biomarkers such as cortisol and creatine kinase, there is a growing need to identify more specific markers for assessing the body’s adaptation to exercise and monitoring training loads. MicroRNAs have emerged as promising candidates in this regard.

The expression of miRNAs changes in response to various physiological or pathological conditions, such as inflammation, cancer, cardiovascular disease, muscle hypertrophy and remodeling, or exercise. In skeletal muscle, miRNAs are involved in the response and adaptation to different modes of exercise, as well as in satellite cell biology, muscle regeneration, and myopathies [51]. Research has shown that miRNAs play a crucial role in the body’s adaptation to exercise, and their levels can fluctuate depending on the type of exercise and the individual’s fitness level [52,53]. Strength training leads to hypertrophic responses, while aerobic exercise increases endurance [15]. Comprehending the pathways and mechanisms that regulate skeletal muscle exercise adaptation is important for understanding athletic performance, fitness, and improving the conduct of more effective training regimes. Several miRNAs regulating skeletal muscle cell metabolism, activation, myogenesis, and differentiation have already been described, indicating a potential role for these miRNAs in skeletal muscle development and hypertrophy [54,55]. These molecules, termed myomiRs, refer to the miRNAs found most abundantly in muscle tissue that act as modulators of skeletal and cardiac muscle proliferation, metabolism, and hypertrophy. The myomiR family includes miR-1, miR-133a, miR-133b, miR-206, miR-208a, miR-208b, miR-486, and miR-499 [42]. Most of them are expressed in both heart and skeletal muscle; however, miR-208a has been identified as cardiovascular-specific and miR-206 as skeletal muscle-specific [51]. In addition to the miRNAs found in the myomiR family, many different miRNAs are more or less involved in muscle function regulation, including miR-24, miR-29, miR-125, miR-181, miR-214, miR-221/222, miR-322/424, miR-503, miR-675, and many others [30,31].

In our study, we focused on determining miRNA-17, miRNA-22, miRNA-24, miRNA-26a, miRNA-93, and miRNA-125b. While they are not all members of the myomiR family, each plays a regulatory role in myogenesis, muscle metabolism, and the inflammatory response. Skeletal muscle is a highly plastic organ. Under the influence of external stimuli, such as nutrition, mechanical loads, or neuromuscular activity, changes in the muscle phenotype occur. Multiple signaling pathways are responsible for muscle development [35,56]. Currently, we are increasingly learning about miRNAs that target a wide range of muscle genes to coordinate the control of the myogenic process [55,57,58]. Some ubiquitously expressed miRNAs also have myogenic functions, and their expression levels typically change during myoblast differentiation. Among others, it has been shown that the expression of miR-26a, as well as miR-24, is increased during myoblast differentiation [59]. The upregulation of miR-24 follows the de-repression from transforming growth factor β (TGFβ)-Smad signaling [60]. De-repression from suppressive signals appears to be a common theme in this subset of myogenic miRNAs, whose upregulation correlates with myogenic differentiation [30,59,60]. MiR-24 and miR-22 are also involved in aging [61]. A study comparing the skeletal muscle biopsies from young and older healthy men showed that age affects microRNA expression. Reduced levels of miR-22 and miR-24 were observed in older men compared to young subjects. It has also been noted that, if miR-24 is overexpressed in primary cardiomyocytes, this can lead to cardiac hypertrophy [62].

Some miRNAs associated with myogenic functions have also been observed to be downregulated. Such miRNAs, including miR-125b, have been identified as suppressors of myogenic differentiation and muscle regeneration [63,64]. The miR-125b expression at the transcriptional level is negatively controlled by mammalian target of rapamycin (mTOR) signaling in an mTOR kinase-independent manner [64]. MiR-125 exists as two isoforms; however, miR-125b appears to play a dominant role in the negative regulation of skeletal myogenesis, while the miR-125a levels in both myoblasts and myotubes are very low [64,65,66]. From these two subtypes of miR-125, both miR-125a and miR-125b are highly expressed in mouse brains, but only miR-125b is also readily detectable in other tissues, including heart, lung, spleen, and skeletal muscle [66]. The researchers noted that the miR-125b levels decrease during myogenesis. MiR-125b does not regulate myoblast proliferation or survival but negatively modulates the myoblast differentiation in the culture. In addition, miR-125b was found to negatively affect the muscle regeneration capacity in mice [64]. Several studies have proven that, during myoblast differentiation, miR-24 and miR-26a, which are considered promoters of myogenesis, increase in abundance [59,60], while miR-125b, which is responsible for inhibiting differentiation and muscle regeneration, decreases [64]. These findings were not corroborated in our study as each miRNA examined exhibited opposite expression patterns. However, a study by Davidsen et al. that investigated the muscle adaptation after a 12-week strength training regimen found that, similar to our results, the miR-26a levels were reduced, while no changes were observed for miR-22, miR-24, or miR-125 [42]. Moreover, miR-125b showed a moderate positive correlation with the CK levels, which may indicate the involvement in the same regulatory patterns of muscle regulation.

We also observed significantly reduced levels of miR-17 after the build-up period. Genetically, the miR-17 gene is located in the polycistronic cluster miR-17-92 [67]. The dysregulation of this microRNA cluster has been associated with skeletal deformities and related growth defects in humans [68,69]. In vivo studies in mice have shown an anti-inflammatory and anti-erosive role of miR-17. Furthermore, miR-17 transfection leads to a reduction in pro-inflammatory cytokines such as IL-6 [70]. In studies on rats, miR-17-5p has been identified as a key factor promoting pathological myocardial hypertrophy, inhibiting *Mfn2* expression, activating the PI3K/AKT/mTOR pathway, and inhibiting autophagy, which leads to heart failure [58]. Zhang and colleagues noted that miR-17 is involved in maintaining cartilage homeostasis and preventing degenerative diseases [36]. Based on their study, they found reduced miR-17 expression in chondrocytes with osteoarthritis, and its deficiency contributed to the progression of osteoarthritis [36]. Aerobic training promotes physiological myocardial hypertrophy and can contribute to preserving cardiac function, counteracting the deterioration of the cardiac function in both damaged and aging hearts [71]. Physiological hypertrophy is induced by numerous humoral factors, as well as mechanical stress, leading to changes in intracellular signaling that affect gene transcription, translation, protein modification, and metabolism [72]. These intracellular changes differ from those observed in pathological hypertrophy. The regulation of the pathways responsible for exercise-induced physiological adaptation has also been shown to occur through microRNAs. Furthermore, increased miR-17 expression is observed in a variety of cancers, including lung, liver, breast, pancreatic, and gastric cancer [73]. The results of our study might indicate that prolonged physical activity reduces the circulating miR-17 levels, which can prevent pathological myocardial hypertrophy. This finding is also supported by the decrease in miR-24 levels. However, the detailed role of miR-17 in muscle function regulation remains to be elucidated.

A bioinformatic analysis revealed that both miRNAs (miR-24 and miR-26a), which appear to be the most promising candidates for the biomarkers of body adaptation to exercise, do not directly regulate the creatine kinase gene muscle subtype. However, they are known to regulate multiple target genes. For instance, miR-24 may potentially bind to the 3′UTR region of the *MAPK14* gene, which encodes the kinase p38α. The functional analysis, based on a mouse model of skeletal muscle regeneration, indicated that miR-24-3p may regulate skeletal muscle fiber-type transformation by controlling the expression of genes such as *Mapk14, Nek4, Pskh1, Pim1,* and *Nlk*. In contrast, other studies demonstrated that myoblasts treated with the p38α/β inhibitor SB203580 ceased the formation of myotubes and the expression of markers of both early and late myogenesis, such as myogenin and creatine kinase [74,75]. Regarding miR-26a, the bioinformatics analysis identified several genes whose expression could potentially be regulated by this molecule, although direct associations were not found. Further investigation and functional analysis are necessary to understand the role of the predicted genes. On another note, previous research has shown that the overexpression of miR-26a in murine myogenic C2C12 cell lines increased the creatine kinase activity during myogenesis. Furthermore, the overexpression of miR-26a resulted in the downregulation of the histone methyltransferase named Enhancer of Zeste homolog 2 (Ezh2), which acts as a suppressor of skeletal muscle cell differentiation [59]. Another study demonstrated Ezh2 degradation via the activation of the p38α cascade [76], which, as mentioned earlier, is regulated by miR-24. Therefore, understanding the miRNA–mRNA–protein relationship network is complex and requires thorough analysis. Our study indicated that both miRNAs may influence the creatine kinase levels and the entire process of myogenesis.

It should be emphasized that our study focused on examining the correlation between the circulating microRNA molecules and the creatine kinase released into the serum. Further research on the influence of these miRs on the expression of the gene encoding creatine kinase is needed to better understand the described relationships.

Our study provides new insights into the human body’s adaptation at the molecular level in response to training, marking a significant stride in comprehending this process. Nonetheless, we acknowledge several limitations, including a relatively small participant pool, a specific profile of the study group, the restriction to one sex, and a diverse set of exercises (aerobic and resistance). Additionally, the lack of similar studies in this area complicates the data interpretation. On the other hand, a strength of the study is that we were able to examine the adaptation process of the body to the exercise of well-trained professional athletes.

## 4. Materials and Methods

### 4.1. Subjects and Study Design

Twelve professional female volleyball players, who are multiple medalists of national and international competitions, participated in this study. The players had a mean age of 27 ± 5.4 years (mean ± SD), an average height of 184.61 cm ± 9.37 cm, and an average body mass of 76.27 kg ± 12.76 kg at baseline. On average, the participants had been training for 13.8± 6.5 years. They did not take any medication, and no injuries were reported during the study period. The Ethics Committee at the University of Rzeszow approved the study (protocol number 3/11/2017), and all individuals provided written informed consent before any procedures. The characterization of the training cycle was previously described [77]. In brief, a 10-week training plan was implemented, comprising 2 introductory weeks, followed by a 6-week preparatory period, and concluding with 2 weeks of specialized training. In total, 1320 min over 11 time units were allocated for training. Maximum oxygen consumption (VO2max), creatine kinase, and cortisol levels in the blood were regularly monitored, along with body composition parameters. VO2max was calculated using results obtained from the BeepTest [78]. Body composition analysis was conducted using the Tanita BC-418 MA analyzer (Tanita Corporation, Tokyo, Japan). Cortisol and creatine kinase were determined using the Alinity analyzer (Abbott, Abbott Park, IL, USA) through the chemiluminescent microparticle immunoassay method and Alinity creatine kinase reagent kit, respectively. Body composition parameters, such as basic metabolic rate (BMR), fat mass (%), fat-free mass (FFM), and total body water (TBW), underwent changes during the training periods and are presented in Table 1.

Samples were collected in the morning after fasting at the beginning (baseline) and end of the 10-week training cycle. Whole blood samples from the median cubital vein were collected in 2 mL tubes with EDTA as an anticoagulant and centrifuged at 2500 rpm for 10 min at room temperature to obtain the plasma. After that, samples were stored at −80 °C until molecular analysis. Subsequently, samples were used to determine the level of circulating extracellular microRNAs.

### 4.2. Circulating MicroRNAs Analysis and Serum Interleukin 6 Determination

Micro-RNAs quantification (miR-22, miR-17, miR-125b, miR-24, miR-26a, and miR-93) and IL-6 levels were measured at two time points: at week 1 (baseline) and week 10 (endpoint). After thawing, plasma samples were centrifuged at 3000× *g* for 5 min to pellet the cells debris and insoluble components. MiRs were extracted from 200 µL of plasma using miRNeasy Serum/Plasma Advanced Kit (Qiagen, Hilden, Germany) according to the manufacturer’s instructions. Total extracted RNAs were reverse transcribed using the miRCURY LNA RT Kit (Qiagen, Hilden, Germany). The detailed procedure was described previously [77]. Subsequently, complementary DNA (cDNA) was stored at −20 °C until further evaluation. Prior to quantitative polymerase chain reaction (qPCR), the cDNA was diluted 30-fold, and the reaction was carried out using SG qPCR Master Mix (EURx, Poland) in the QuantStudio real-time PCR system (Applied Biosystems, Waltham, MA, USA). The PCR was performed under the following thermal cycling conditions: 95 °C for initial denaturation, 94 °C for 15 s for denaturation, 60 °C for 30 s for annealing, and 72 °C for 30 s for extension. The reaction was carried out for 45 amplification cycles, and all samples were evaluated in duplicates. Both mimic cel-miR-39-3p and hsa-miR-425-5p were used for expression normalization. The primer sequences were previously described [79], except for those complementary to miR-425 and hsa-miR-24-3p, which were designed using the miRprimer v.2 software [80]. The sequences were as follows (5′→3′): forward: GGCAGAATGACACGATCACTCC, reverse: GGTCCAGTTTTTTTTTTTTTTTCAAC and forward: AGCAGTGGCTCAGTTCAGCA, reverse: ACCAG-TTTTTTTTTTTTTTTCTGTTC, respectively. All primers were used at a final concentration of 450 nM. Data were collected and analyzed using the QuantStudio Design and Analysis Software v1.5.2 (Applied Biosystems, USA). The comparative Ct method (ΔΔCt method) was employed to evaluate miRs quantification, and the expression results were calculated as relative quantitation (RQ) and presented as relative fold change. A calibrator sample, prepared by mixing the cDNA of randomly selected samples, was used as the reference (control). The cDNA was aliquoted and frozen to avoid repeated thawing, and it was added to each reaction plate to normalize miRNA quantities and eliminate inter-plate variability. Interleukin-6 (IL6) levels were determined in serum using an enzyme-linked immunosorbent immunoassay (ELISA) kit (Interleukin-6 Human ELISA kit, BioVendor R&D, cat no. RD194015200R) and an absorbance reader (Tecan infinite M200 Pro reader and Magellan software, version 7.1). All procedures were conducted according to the manufacturer’s recommendations, and standards and samples were evaluated in duplicates.

### 4.3. Statistical Analysis

Quantitative values with normal distribution were presented as mean ± SD or median (lower–upper quartile). Depending on the distribution, as assessed by the Shapiro–Wilk W test, differences between the two training periods were compared using the dependent *t*-test for paired samples; otherwise, the Wilcoxon test was applied. The plasma relative quantity of miR-17, miR-125b, miR-24, miR-26a, and IL-6 concentrations was transformed using the logarithm function to achieve a normal distribution. The relationships between continuous variables with normal distribution were analyzed using Pearson’s correlation; otherwise, Spearman’s rank correlation was utilized. Multiple linear regression analysis was conducted to estimate the impact of miR-22, miR-17, miR-24, and miR-26a on the levels of creatine kinase, cortisol, as well as VO2 max. A *p*-value less than 0.05 was considered statistically significant. The analysis was performed using STATISTICA Version 13.3 (Dell Inc., 2016, Tulsa, OK, USA).

### 4.4. Bioinformatic Analysis

The interactions between miRs-24 and miR-26a and messenger RNAs (mRNAs) were tested in silico using the online tool miRWalk, available from http://mirwalk.umm.uni-heidelberg.de (accessed on 8 May 2024) [81]. The following filtering parameters were applied: minimum binding probability 0.95, binding site 3′UTR, as well as validated interactions; the results with the interactions of Targetscan [82,83] and the Mirdb database [84] and Mirtarbase [85] were considered for further analysis. 

## 5. Conclusions

In our study, we investigated the effects of intensive training on circulating microRNA levels and their correlation with the established physical activity markers in elite female volleyball players. Our results reveal changes in specific microRNAs, such as miR-17, miR-22, miR-24, and miR-26a, over a 10-week training period, which might be linked to the body’s response to inflammation. Notably, we observed a significant decrease in these microRNAs after training, along with reduced levels of inflammatory biomarkers such as cortisol and IL-6, suggesting a potential link between microRNAs and physiological adaptations to exercise. Particularly noteworthy was the relationship between decreased miR-24 and miR-26a levels and elevated creatine kinase, indicating their potential involvement in the muscle repair processes. Additionally, significant changes in the body composition parameters underscored the impact of training on various physiological aspects. Intense physical activity usually increases the release of inflammatory biomarkers, which was not observed in our study, probably due to the better adaptation of the professional athletes’ bodies to training loads as well as their proper planning to avoid inducing a negative inflammatory response. Our findings suggest that the investigated microRNAs, especially miR-24 and miR-26a, could serve as biomarkers reflecting the body’s adaptive response to exercise.

## Figures and Tables

**Table 1:** Differences in body composition parameters between study periods.

Body Composition Parameter	Baseline	Endpoint	*p*-Value
Mass, kg	77.3 ± 10.08	78.17 ± 10.49	0.070
BMI, kg/m^2^	22.68 ± 2.18	22.85 ± 2.18	0.210
Fat, %	20.83 ± 3.77	18.66 ± 3.58	**<0.001**
Fat mass, kg	16.33 ± 4.76	14.89 ± 4.7	**0.003**
FFM, kg	60.98 ± 6.38	63.53 ± 6.85	**0.002**
TBW, kg	44.63 ± 4.66	46.45 ± 5.05	**0.002**
BMR, kJ	7568.75 ± 848.73	7828.5 ± 910.59	**0.002**
VO2max, mL/kg/min.	41.8 ± 4.54	45.57 ± 4.69	**<0.001**

Body composition data are presented as mean ± SD. Abbreviations: BMI, body mass index; BMR, basic metabolic rate; FFM, fat-free mass; TBW, total body water. A *p*-value was estimated using the dependent *t*-test for paired samples or the Wilcoxon test. Statistically significant differences are indicated in bold.

**Table 2:** Differences in the levels of the targeted markers and laboratory parameters between study periods.

Parameter	Baseline	Endpoint	*p*-Value
miR-17	0.35 ± 0.13	0.23 ± 0.08	**0.039**
miR-22	0.40 ± 0.1	0.28 ± 0.12	**0.009**
miR-24	0.09 ± 0.04	0.05 ± 0.02	**0.001**
miR-26a	0.11 ± 0.06	0.06 ± 0.04	**0.003**
miR-93	0.36 ± 0.1	0.36 ± 0.12	0.980
miR-125b	0.22 ± 0.06	0.28 ± 0.1	0.185
IL-6 [pg/mL] Norm < 7	4.11 ± 0.76	3.64 ± 0.68	0.220
CK [U/L] Reference range [29–168]	101.82 ± 51.12	210.58 ± 80.91	**0.001**
Cortisol [µg/dL] Reference range [3.70–19.40]	25.57 ± 7.45	16.66 ± 4.26	**<0.001**

MicroRNAs’ quantity values are presented as relative fold change and provided as mean ± SD or median [lower–upper quartile]. Abbreviations: CK, creatine kinase; miR, micro-RNA. A *p*-value was estimated by repeated measures ANOVA. Statistically significant differences were bolded.

**Table 3:** Endpoint correlation between micro-RNAs’ relative quantity and body composition as well as laboratory parameters.

Parameter	Weight	BMI	BMR	FAT%	Fat Mass	FFM	TBW	VO2 Max	Creatine Kinase	Cortisol	IL-6
miR-22	0.002	−0.10	−0.09	0.22	0.14	−0.11	−0.09	0.01	0.22	0.57	−0.17
miR-17	−0.17	−0.52	−0.03	−0.48	−0.44	0.03	0.03	0.24	−0.28	0.31	0.33
miR-125b	−0.09	0.35	−0.24	0.35	0.25	−0.3	−0.3	0.19	0.53	0.04	0.16
miR-24	0.13	**0.63**	0.08	0.17	0.23	0.03	0.03	0.24	**0.67**	−0.24	0.35
miR-26a	−0.06	0.47	−0.04	−0.08	−0.03	−0.09	−0.08	0.4	0.46	−0.16	0.4
miR-93	−0.02	−0.32	0.05	−0.26	−0.2	0.11	0.10	0.04	−0.08	0.26	0.27

Abbreviations: please refer to Table 1. Statistically significant differences were bolded.

**Table 4:** The multilinear logistic regression analysis using creatine kinase concentration as outcome.

The creatine kinase concentration was used as dependent variable. R = 0.9; R2 = 0.8 and R2 (adjusted) = 0.69						
**micro-RNA name**	b *	Standard Error from b *	**b**	**Standard Error from b**	**T**	***p*** -Value *
**Intercept**			−52.22	71.468	−0.731	0.489
**miR-22**	0.169	0.178	117.130	123.500	0.948	0.375
**miR-17**	0.233	0.174	223.570	166.679	1.341	0.222
**miR-24**	1.563	0.343	6218.670	1364.871	4.556	**0.003**
**miR-26a**	−0.861	0.347	−1715.980	691.530	−2.481	**0.042**

* Statistically significant differences were bolded.

## Data Availability

Data is contained within the article and Supplementary Materials.

## Supplementary Materials

The following supporting information can be downloaded at https://www.mdpi.com/article/10.3390/ijms25116107/s1.
