# Supplementary material for: Changes in Circulating MicroRNA Levels as Potential Indicators of Training Adaptation in Professional Volleyball Players
Source: Int J Mol Sci. 2024 Jun 1;25(11):6107. doi: 10.3390/ijms25116107 (PMC11173131; doi:10.3390/ijms25116107)
Supplement: Supplementary file 1 [file ijms-25-06107-s001.zip › ijms-2993175-supplementary.pdf]

Supplementary file.

Table S1. Whole training period correlation between micro-RNAs relative quantity and body composition as well as laboratory parameters.

	Weigh	BMI	BMR	FAT%	Fat mass	FFM	TBW	VO_2 max	Creatine kinase	Cortisol	IL-6
**miR-22**	-0.01	-0.03	-0.07	0.22	0.14	-0.12	-0.11	-0.13	-0.33	0.33	-0.03
**miR-17**	-0.10	-0.16	-0.05	-0.13	-0.15	-0.06	-0.05	-0.11	-0.39	0.19	0.09
**miR-125b**	-0.22	0.05	-0.29	0.08	0.01	-0.31	-0.31	0.22	**0.46**	-0.10	-0.11
**miR-24**	0.05	0.34	-0.05	0.29	0.25	-0.11	-0.10	-0.23	-0.11	0.23	0.22
**miR-26a**	-0.13	0.25	-0.18	0.09	0.02	-0.23	-0.22	-0.06	-0.17	0.39	0.28
**miR-93**	-0.08	-0.32	0.01	-0.34	-0.27	0.05	0.05	0.06	0.05	0.01	0.07

Abbreviations: BMI, body mass index; BMR, basic metabolic rate; FFM, fat free mass; IL-6, interleukin 6; TBW, total body water. Significant correlation is bold and indicated in red.

Table S2. Baseline period correlation between micro-RNAs relative quantity and body composition as well as laboratory parameters.

	**Weigh**	**BMI**	**BMR**	**FAT%**	**Fat mass**	**FFM**	**TBW**	**VO_2 max**	**Creatine kinase**	**Cortisol**	**IL-6**
**miR-22**	0.05	0.12	0.14	-0.10	-0.04	0.10	0.10	0.11	-0.29	-0.38	-0.37
**miR-17**	0.01	0.2	0.10	-0.21	-0.08	0.07	0.07	0.05	0.12	-0.48	-0.54
**miR-125b**	-0.49	-0.45	-0.54	-0.09	-0.26	-0.54	-0.54	0.19	0.13	0.15	-0.33
**miR-24**	0.09	0.32	0.05	0.11	0.17	0.01	0.01	-0.19	0.38	-0.29	-0.31
**miR-26a**	-0.27	0.13	-0.26	-0.16	-0.18	-0.27	-0.27	0.04	0.07	0.32	-0.39
**miR-93**	-0.18	-0.33	-0.04	-0.47	-0.37	-0.01	-0.01	0.23	0.26	-0.16	-0.15

Abbreviations: Please refer to Table S1.

Table S3. The multilinear logistic regression analysis using cortisol concentration as outcome.

The cortisol concentration was used as dependent variable. $R = 0.67$; $R^2 = 0.45$ and R^2 (adjusted) = 0.14						
**micro-RNA name**	b*	Standard error from b*	b	Standard error from b	T	p-value
**Intercept**			5.9604	6.2991	0.946231	0.375540
**miR-22**	0.589616	0.297361	21.5834	10.8852	1.982828	0.087815
**miR-17**	0.373014	0.290160	18.8859	14.6910	1.285547	0.239494
**miR-24**	-0.118220	0.573726	-24.7883	120.2987	-0.206057	0.842612
**miR-26a**	0.200736	0.580047	21.0931	60.9509	0.346068	0.739460

Table S4. The multilinear logistic regression analysis using VO_2 max as outcome.

The VO_2 max was used as dependent variable.<br>$R = 0.65$; $R^2 = 0.43$ and R^2 (adjusted) = 0.097						
**micro-RNA name**	b*	Standard error from b*	b	Standard error from b	T	p-value
**Intercept**			47.058	7.1033	6.62485	0.000297
**miR-22**	0.20890	0.304770	8.414	12.2749	0.68544	0.515117
**miR-17**	-0.07881	0.297389	-4.390	16.5665	-0.26499	0.798648
**miR-24**	-1.19563	0.588022	-275.834	135.6572	-2.03331	0.081513
**miR-26a**	1.34746	0.594500	155.785	68.7325	2.26654	0.057771

Table S5. Genes potentially regulated by hsa-miR-24-3p.

Gene symbol	Description
DNAJB12	Homo sapiens DnaJ heat shock protein family (Hsp40) member B12(DNAJB12), transcript variant 1, mRNA.
RAB11FIP1	Homo sapiens RAB11 family interacting protein 1 (RAB11FIP1),transcript variant 3, mRNA.
MAGI1	Homo sapiens membrane associated guanylate kinase, WW and PDZdomain containing 1 (MAGI1), transcript variant 3, mRNA.
AGPAT3	Homo sapiens 1-acylglycerol-3-phosphate O-acyltransferase 3(AGPAT3), transcript variant 2, mRNA.
MBOAT1	Homo sapiens membrane bound O-acyltransferase domain containing 1(MBOAT1), transcript variant 1, mRNA.
TSPAN14	Homo sapiens tetraspanin 14 (TSPAN14), transcript variant 2, mRNA.
INAVA	Homo sapiens innate immunity activator (INAVA), transcript variant2, mRNA.
NEK6	Homo sapiens NIMA related kinase 6 (NEK6), transcript variant 1,mRNA.
SESN1	Homo sapiens sestrin 1 (SESN1), transcript variant 2, mRNA.
BCL2L11	Homo sapiens BCL2 like 11 (BCL2L11), transcript variant 11, mRNA.
STRADB	Homo sapiens STE20 related adaptor beta (STRADB), transcriptvariant 2, mRNA.
ABCB9	Homo sapiens ATP binding cassette subfamily B member 9 (ABCB9),transcript variant 6, mRNA.
RAP2C	Homo sapiens RAP2C, member of RAS oncogene family (RAP2C),transcript variant 1, mRNA.
YOD1	Homo sapiens YOD1 deubiquitinase (YOD1), transcript variant 2,mRNA.
GUCD1	Homo sapiens guanylyl cyclase domain containing 1 (GUCD1),transcript variant 1, mRNA.
SSR1	Homo sapiens signal sequence receptor subunit 1 (SSR1), transcriptvariant 2, mRNA.
MLEC	Homo sapiens malectin (MLEC), transcript variant 2, mRNA.
MAPK14	Homo sapiens mitogen-activated protein kinase 14 (MAPK14),transcript variant 1, mRNA.
PTPRF	Homo sapiens protein tyrosine phosphatase receptor type F (PTPRF),transcript variant 3, mRNA.

GBA2	Homo sapiens glucosylceramidase beta 2 (GBA2), transcript variant2, mRNA.
PDGFRB	Homo sapiens platelet derived growth factor receptor beta (PDGFRB),transcript variant 2, mRNA.
AAK1	Homo sapiens AP2 associated kinase 1 (AAK1), transcript variant 2,mRNA.
ZNF217	Homo sapiens zinc finger protein 217 (ZNF217), transcript variant1, mRNA.
CDKN1B	Homo sapiens cyclin dependent kinase inhibitor 1B (CDKN1B), mRNA.
ACVR1B	Homo sapiens activin A receptor type 1B (ACVR1B), transcriptvariant 1, mRNA.
PRKCH	Homo sapiens protein kinase C eta (PRKCH), mRNA.
ABHD2	Homo sapiens abhydrolase domain containing 2, acylglycerol lipase(ABHD2), transcript variant 1, mRNA.
SNTB1	Homo sapiens syntrophin beta 1 (SNTB1), mRNA.
VCPIP1	Homo sapiens valosin containing protein interacting protein 1(VCPIP1), mRNA.
CMTM4	Homo sapiens CKLF like MARVEL transmembrane domain containing 4(CMTM4), transcript variant 1, mRNA.

Table S6. Genes potentially regulated by hsa-miR-26a-5p.

**Gene symbol**	**Description**
PDE4B	Homo sapiens phosphodiesterase 4B (PDE4B), transcript variant b,mRNA.
TET2	Homo sapiens tet methylcytosine dioxygenase 2 (TET2), transcriptvariant 1, mRNA.
CHAC1	Homo sapiens ChaC glutathione specificgamma-glutamylcyclotransferase 1 (CHAC1), transcript variant 2,
ELAVL2	Homo sapiens ELAV like RNA binding protein 2 (ELAVL2), transcriptvariant 2, mRNA.
CPEB3	Homo sapiens cytoplasmic polyadenylation element binding protein 3(CPEB3), transcript variant 2, mRNA.
DDX3X	Homo sapiens DEAD-box helicase 3 X-linked (DDX3X), transcriptvariant 2, mRNA.
HMGA1	Homo sapiens high mobility group AT-hook 1 (HMGA1), transcriptvariant 8, mRNA.
CPSF2	Homo sapiens cleavage and polyadenylation specific factor 2(CPSF2), transcript variant 2, mRNA.

ZNF608	Homo sapiens zinc finger protein 608 (ZNF608), transcript variant2, mRNA.
ABHD2	Homo sapiens abhydrolase domain containing 2, acylglycerol lipase(ABHD2), transcript variant 1, mRNA.
KPNA6	Homo sapiens karyopherin subunit alpha 6 (KPNA6), mRNA.
ZDHHC18	Homo sapiens zinc finger DHHC-type palmitoyltransferase 18(ZDHHC18), mRNA.
